# The Mammoth Cave

**Published:** 1859-04

**Authors:** 


*The Mammoth Cave.* By CHARLES W. WRIGHT, M. D., Professor of Chemistry in the Kentucky School of Medicine.

ATMOSPHERE OF THE CAVE.—The proportions of oxygen and nitrogen bear the same relation to each other in the Mammoth Cave that they do in the external air. The proportion of carbonic acid gas is less than that observed in the atmosphere of the surrounding country, upon an average of many observations. In the dry parts of the cave, the proportion is about 2 to 10,000 of air; in the vicinity of the rivers, something less. Not a trace of ammonia can be detected in those parts of the cave not commonly visited. The amount of the vapor of water varies. Thus, in those avenues, at a great distance from the rivers, upon the walls and floors of which there is a deposit of nitrate of lime, the air is almost entirely destitute of moisture, from the hygroscopic properties of that salt, and animal matter mummifies instead of suffering putrifactive decomposition. And for the same reason, no matter what state of division the disentegrated rock may attain, dust never rises. In portions of the cave remote from the localities in which the bats hybernate, no organic matter can be recognised by the most delicate tests. Not a trace of ozone can be detected by the most sensitive reägents.

From what has been stated, it will be observed that the atmosphere of the Mammoth Cave is free from those substances which are calculated to exert a depressing and septic influence on the animal economy, than that of any other locality of the globe. This great difference is observed by every one on leaving the cave, after having remained in it for a number of hours. In such instances the impurity of the external air is almost insufferably offensive to the sense of smell, and the romance of a "pure country air" is forever dissipated.

What diseases would be benefited, or rendered worse by resorting to the Mammoth Cave?

Consumptives, at one time, resorted to the cave, and, as might have been anticipated, with fatal results. Several of them died there, and all of them, soon after exposure to the external air. One patient did not see the light of the sun for a period of five months. Short trips are attended with advantage, but a cave residence is speedily fatal.

I know of no inflammatory disease that is rendered worse by a resort to the Mammoth Cave. On the contrary, short and easy trips have been known to effect a cure in chronic dysentery and diarrhœa, where all other measures had failed.

In all those diseases where absolute silence, and the total exclusion of light are indicated, the cave above all other places, possesses preëminent advantages; for no where else have we those conditions combined. The only condition in which risk is incurred is during the menstrual period. Serious, and even fatal, results have been the consequence of inattention to this fact.

The temperature of the Mammoth Cave is uniformly 59 degrees, winter and summer; which, in connection with the remarkable purity of its atmosphere, will account for the fact that individuals are enabled to undergo such an unusual amount of physical exertion in it. It is not an uncommon occurrence for a person in delicate health to accomplish a journey of twenty miles in the cave, without suffering from fatigue, who could not be prevailed upon to walk a distance of three miles on the surface of the earth.—*Louisville Medical Gazette.*

---

*M. Falcony's Powder for Preserving Dead Bodies.*—The result of a very successful trial of M. Falcony's mode of preserving dead bodies was seen at the Grosvenor-place School on Tuesday. A body was brought to the school on the 24th of September, in a state of decomposition, so advanced, as to be quite unfit for dissection. It was covered with M. Falcony's powder, and left in an open coffin until Tuesday last, when it was inspected in the presence of a number of scientific and literary men. There had not been the least offensive odcr in the room in which the coffin was kept, and the body on Tuesday was quite free from odor. The powder used contains a large proportion of dried sulphate of zinc; this is mixed with common sawdust of white pine, before covering the body. The rationale of the process is easily understood. The sawdust keeps the oxygen of the atmosphere from access to the body, and the emanations from the body are oxydized in the sawdust by the atmospheric oxygen. Hence there is no escape of the fetid gases. Then internal decomposition is prevented by the sulphate of zinc absorbing the water of the body, deliquescing, and recrystalizing as hydrate, probably with seven equivalents of water of crystalization. Whether this explanation be correct or not, there can be no doubt whatever that the process is a cheap, safe, and effectual one, perfectly fulfilling its intended object; and we feel sure that the adoption of such a process with all our dead, would tend to protect the living from cadaveric poisoning of the air we breathe and the water we drink.—*Med. Times and Gazette, Oct. 23d,* 1858, from *The N. A. Medico-Chirurgical Review.*

